# (*RS*)-Dimethyl­ammonium 2-*sec*-butyl-4,6-dinitro­phenolate

**DOI:** 10.1107/S1600536809038677

**Published:** 2009-10-03

**Authors:** Carl Henrik Görbitz

**Affiliations:** a Department of Chemistry, University of Oslo, PO Box 1033 Blindern, N-0315 Oslo, Norway

## Abstract

The title compound, C_2_H_8_N^+^·C_10_H_11_N_2_O_5_^−^, is a highly toxic herbicide known as dinoseb. The *sec*-butyl group is disordered [occupancy ratio 0.828 (3):0.172 (3)], while the nitro group in the 6 position is twisted by 25° with respect to the ring plane. Pairs of –O^−^⋯H—N^+^—H⋯^−^O– bridges between phenolic O atoms generate eight-membered hydrogen-bonded rings.

## Related literature

For toxicicity information, see: EXTOXNET (1996 ▶). Related structures have been described by Smith *et al.* (2002 ▶, 2005 ▶); Lynch & McClenaghan (2004 ▶); West-Nielsen *et al.* (2006 ▶). 
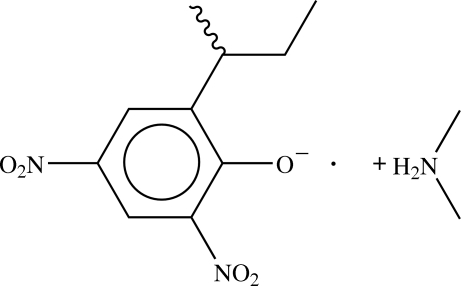


## Experimental

### 

#### Crystal data

C_2_H_8_N^+^·C_10_H_11_N_2_O_5_^−^
*M* *_r_* = 285.30
Monoclinic, 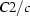

*a* = 16.804 (4) Å
*b* = 9.1446 (17) Å
*c* = 19.223 (4) Å
β = 104.555 (6)°
*V* = 2859.2 (10) Å^3^
*Z* = 8
Mo *K*α radiation
μ = 0.10 mm^−1^
*T* = 105 K
0.70 × 0.09 × 0.05 mm

#### Data collection

Siemens SMART CCD diffractometer
Absorption correction: multi-scan (*SADABS*; Sheldrick, 1996 ▶) *T*_min_ = 0.832, *T*_max_ = 0.995
10444 measured reflections
2909 independent reflections
2382 reflections with *I* > 2σ(*I*)
*R*_int_ = 0.026

#### Refinement

*R*[*F* ^2^ > 2σ(*F* ^2^)] = 0.034
*wR*(*F* ^2^) = 0.094
*S* = 1.05
2909 reflections
219 parameters
45 restraints
H atoms treated by a mixture of independent and constrained refinement
Δρ_max_ = 0.21 e Å^−3^
Δρ_min_ = −0.20 e Å^−3^

### 

Data collection: *SMART* (Bruker, 1998 ▶); cell refinement: *SAINT-Plus* (Bruker, 2001 ▶); data reduction: *SAINT-Plus* ; program(s) used to solve structure: *SHELXTL* (Sheldrick, 2008 ▶); program(s) used to refine structure: *SHELXTL*; molecular graphics: *SHELXTL* software used to prepare material for publication: *SHELXTL*.

## Supplementary Material

Crystal structure: contains datablocks I, global. DOI: 10.1107/S1600536809038677/pk2194sup1.cif

Structure factors: contains datablocks I. DOI: 10.1107/S1600536809038677/pk2194Isup2.hkl

Additional supplementary materials:  crystallographic information; 3D view; checkCIF report

## Figures and Tables

**Table 1:** Hydrogen-bond geometry (Å, °)

*D*—H⋯*A*	*D*—H	H⋯*A*	*D*⋯*A*	*D*—H⋯*A*
N1*B*—H1*B*⋯O1*A*	0.904 (16)	1.914 (16)	2.7585 (15)	154.7 (14)
N1*B*—H2*B*⋯O1*A*^i^	0.902 (17)	1.868 (17)	2.7173 (16)	156.2 (15)

Symmetry code: (i) 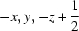.

**Fig. 1.**
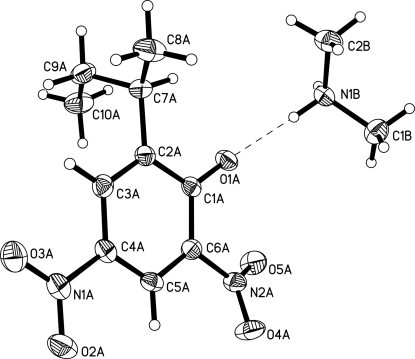
: The molecular structure of (I) (major disorder component). Displacement ellipsoids are shown at the 50% probability level and H-atoms are shown as spheres of arbitrary size.

**Fig. 2.**
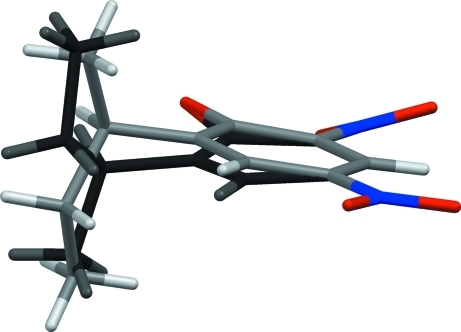
: Disorder for the sec-butyl group and neighbouring atoms. Normal, light colour is used for the major component with R-configuration at C7, the minor component with S-configuration appears in darker colour.

## supplementary crystallographic information

### Comment

Large amounts of a highly volatile yellow powder of unknown origin was discovered below the floor planks in the attic of a private home, and a small quantity was forwarded to the University of Oslo for analysis. Single crystals were easily obtained, and subsequent structure determination by X-ray diffraction identified the sample as a dimethylammonium salt of 2-*sec*-butyl-4,6-dinitrophenol, with common name dinoseb (I). This herbicide has been used in soybeans, vegetables, fruits and nuts, citrus, and other field crops for the selective control of grass and broadleaf weeds (*e.g.*, in corn). It was also used as an insecticide in grapes and as a seed crop drying agent. Product names for pesticides containing dinoseb include Basanite, Caldon, Chemox, Chemsect DNBP, Dinitro, Dynamyte, Elgetol, Gebutox, Hel-Fire, Kiloseb, Nitropone, Premerge, Sinox General, Subitex, and Vertac Weed Killer. The use of dinoseb was prohibited in the U.S. in 1986, an action based on the potential risk of birth defects and other adverse health effects (EXTOXNET, 1996).

2-*sec*-butyl-4,6-dinitrophenol has a chiral C-atom at C7, and the title salt is racemic, space group *C*2/c. For simplicity, the *R*-form alone is shown in Fig. 1, but there is actually disorder at each phenolate position giving a 0.828 (3):0.172 (3) distribution between the *R*-form and the S-form, as in Fig. 2, or *vice versa*. Both ammonium H-atoms form strong hydrogen bonds to charged phenolate O atom acceptors, one interaction is visible in Fig. 1.

Related structures include achiral analogues with *tert*-butyl (Lynch & McClenaghan, 2004) and acetyl (= 2-hydroxy-3,5-dinitroacetophenone; West-Nielsen *et al.*, 2006) substituents rather than *sec*-butyl at C2 (both obtained as neutral molecules) as well as a series of proton-transfer complexes of 3,5-dinitrosalicylic acid with aliphatic amines (Smith *et al.*, 2002; Smith *et al.*, 2005).

### Experimental

Crystals in the shape of fine, yellow needles were grown by slow evaporation of a diethylether solution of the title complex.

### Refinement

Normal anisotropic refinement except for atoms with low occupancy (minor disorder component), which were either refined isotropically (*sec*-butyl group) or constrained to have the same thermal parameters as the near-by atom of the major disorder component (C1, C2, C3). The geometries of the minor and the major component, in terms of covalent bond lengths and bond angles, were restrained to be more or less similar by a *SHELXTL* SAME command. Positional parameters were refined for the two ammonium H atoms involved in hydrogen bonding, other H atoms were positioned with idealized geometry and C–H distances fixed in the range 0.95 to 0.99 Å. *U*_iso_ values were 1.2*U*_eq_ of the carrier atom, or 1.5*U*_eq_ for the methyl groups.

### Crystal data

**Table:**

C_2_H_8_N^+^·C_10_H_11_N_2_O_5_^−^	*F*(000) = 1216
*M**_r_* = 285.30	*D*_x_ = 1.326 Mg m^−^^3^
Monoclinic, *C*2/*c*	Mo *K*α radiation, λ = 0.71073 Å
*a* = 16.804 (4) Å	Cell parameters from 4501 reflections
*b* = 9.1446 (17) Å	θ = 2.2–26.4°
*c* = 19.223 (4) Å	µ = 0.10 mm^−^^1^
β = 104.555 (6)°	*T* = 105 K
*V* = 2859.2 (10) Å^3^	Needle, yellow
*Z* = 8	0.70 × 0.09 × 0.05 mm

### Data collection

**Table:**

Siemens SMART CCD diffractometer	2909 independent reflections
Radiation source: fine-focus sealed tube	2382 reflections with *I* > 2σ(*I*)
graphite	*R*_int_ = 0.026
Detector resolution: 8.3 pixels mm^-1^	θ_max_ = 26.4°, θ_min_ = 2.2°
ω scans	*h* = −20→20
Absorption correction: multi-scan (*SADABS*; Sheldrick, 1996)	*k* = −11→11
*T*_min_ = 0.832, *T*_max_ = 0.995	*l* = −23→22
10444 measured reflections	

### Refinement

**Table:**

Refinement on *F*^2^	Primary atom site location: structure-invariant direct methods
Least-squares matrix: full	Secondary atom site location: difference Fourier map
*R*[*F*^2^ > 2σ(*F*^2^)] = 0.034	Hydrogen site location: inferred from neighbouring sites
*wR*(*F*^2^) = 0.094	H atoms treated by a mixture of independent and constrained refinement
*S* = 1.05	*w* = 1/[σ^2^(*F*_o_^2^) + (0.0469*P*)^2^ + 1.2758*P*] where *P* = (*F*_o_^2^ + 2*F*_c_^2^)/3
2909 reflections	(Δ/σ)_max_ = 0.002
219 parameters	Δρ_max_ = 0.21 e Å^−^^3^
45 restraints	Δρ_min_ = −0.20 e Å^−^^3^

### Special details

**Table:**

Experimental. Crystallized from diethyl ether.Three sets of frames each taken over 0.3° ω rotation with 20 s exposure time. Detector set at 2θ = 27°, crystal-to-detector distance 5.00 cm.
Geometry. All e.s.d.'s (except the e.s.d. in the dihedral angle between two l.s. planes) are estimated using the full covariance matrix. The cell e.s.d.'s are taken into account individually in the estimation of e.s.d.'s in distances, angles and torsion angles; correlations between e.s.d.'s in cell parameters are only used when they are defined by crystal symmetry. An approximate (isotropic) treatment of cell e.s.d.'s is used for estimating e.s.d.'s involving l.s. planes.
Refinement. Refinement on *F*^2^ against ALL reflections.

### Fractional atomic coordinates and isotropic or equivalent isotropic displacement parameters (Å^2^)

**Table:**

	*x*	*y*	*z*	*U*_iso_*/*U*_eq_	Occ. (<1)
O1A	0.09291 (5)	0.27723 (10)	0.31483 (5)	0.0280 (2)	0.828 (3)
O2A	0.43766 (6)	0.16711 (12)	0.52414 (6)	0.0379 (3)	0.828 (3)
O3A	0.37950 (6)	−0.04279 (12)	0.52952 (6)	0.0417 (3)	0.828 (3)
O4A	0.27707 (6)	0.55996 (11)	0.39749 (6)	0.0391 (3)	0.828 (3)
O5A	0.18713 (6)	0.50849 (11)	0.29870 (6)	0.0372 (3)	0.828 (3)
N1A	0.37863 (7)	0.08255 (13)	0.50556 (6)	0.0289 (3)	0.828 (3)
N2A	0.23146 (7)	0.47265 (12)	0.35751 (6)	0.0272 (3)	0.828 (3)
C1A	0.15919 (8)	0.23247 (15)	0.35793 (10)	0.0211 (4)	0.828 (3)
C2A	0.16612 (9)	0.08529 (15)	0.38681 (9)	0.0229 (4)	0.828 (3)
C3A	0.23721 (9)	0.03893 (16)	0.43364 (10)	0.0248 (4)	0.828 (3)
H31A	0.2404	−0.0576	0.4524	0.030*	0.828 (3)
C4A	0.30549 (7)	0.13271 (15)	0.45422 (7)	0.0245 (3)	0.828 (3)
C5A	0.30269 (7)	0.27426 (15)	0.42947 (7)	0.0235 (3)	0.828 (3)
H51A	0.3483	0.3380	0.4452	0.028*	0.828 (3)
C6A	0.23184 (8)	0.32142 (14)	0.38106 (7)	0.0234 (3)	0.828 (3)
C7A	0.09301 (9)	−0.01813 (17)	0.36283 (9)	0.0273 (4)	0.828 (3)
H71A	0.0486	0.0358	0.3279	0.033*	0.828 (3)
C8A	0.05870 (16)	−0.0639 (4)	0.42727 (16)	0.0404 (6)	0.828 (3)
H81A	0.0112	−0.1283	0.4104	0.061*	0.828 (3)
H82A	0.0418	0.0235	0.4494	0.061*	0.828 (3)
H83A	0.1014	−0.1157	0.4628	0.061*	0.828 (3)
C9A	0.11577 (11)	−0.15163 (18)	0.32438 (10)	0.0328 (5)	0.828 (3)
H91A	0.1610	−0.2049	0.3577	0.039*	0.828 (3)
H92A	0.0678	−0.2180	0.3114	0.039*	0.828 (3)
C10A	0.1424 (2)	−0.1119 (3)	0.25677 (14)	0.0392 (7)	0.828 (3)
H11A	0.1603	−0.2004	0.2362	0.059*	0.828 (3)
H12A	0.1881	−0.0421	0.2689	0.059*	0.828 (3)
H13A	0.0961	−0.0678	0.2217	0.059*	0.828 (3)
O1C	0.09291 (5)	0.27723 (10)	0.31483 (5)	0.0280 (2)	0.172 (3)
O2C	0.43766 (6)	0.16711 (12)	0.52414 (6)	0.0379 (3)	0.172 (3)
O3C	0.37950 (6)	−0.04279 (12)	0.52952 (6)	0.0417 (3)	0.172 (3)
O4C	0.27707 (6)	0.55996 (11)	0.39749 (6)	0.0391 (3)	0.172 (3)
O5C	0.18713 (6)	0.50849 (11)	0.29870 (6)	0.0372 (3)	0.172 (3)
N1C	0.37863 (7)	0.08255 (13)	0.50556 (6)	0.0289 (3)	0.172 (3)
N2C	0.23146 (7)	0.47265 (12)	0.35751 (6)	0.0272 (3)	0.172 (3)
C1C	0.1637 (2)	0.2281 (3)	0.3484 (4)	0.0211 (4)	0.172 (3)
C2C	0.1743 (2)	0.0761 (3)	0.3713 (3)	0.0229 (4)	0.172 (3)
C3C	0.2435 (3)	0.0322 (3)	0.4213 (4)	0.0248 (4)	0.172 (3)
H31C	0.2499	−0.0682	0.4342	0.030*	0.172 (3)
C4C	0.30549 (7)	0.13271 (15)	0.45422 (7)	0.0245 (3)	0.172 (3)
C5C	0.30269 (7)	0.27426 (15)	0.42947 (7)	0.0235 (3)	0.172 (3)
H51C	0.3483	0.3380	0.4452	0.028*	0.172 (3)
C6C	0.23184 (8)	0.32142 (14)	0.38106 (7)	0.0234 (3)	0.172 (3)
C7C	0.1084 (3)	−0.0307 (4)	0.3324 (3)	0.032 (2)*	0.172 (3)
H7C	0.0566	0.0271	0.3158	0.038*	0.172 (3)
C8C	0.1287 (9)	−0.0929 (13)	0.2640 (5)	0.052 (7)*	0.172 (3)
H81C	0.1447	−0.0128	0.2365	0.078*	0.172 (3)
H82C	0.0802	−0.1424	0.2345	0.078*	0.172 (3)
H83C	0.1741	−0.1629	0.2778	0.078*	0.172 (3)
C9C	0.0903 (4)	−0.1504 (6)	0.3806 (4)	0.035 (2)*	0.172 (3)
H91C	0.1422	−0.2010	0.4038	0.041*	0.172 (3)
H92C	0.0531	−0.2229	0.3507	0.041*	0.172 (3)
C10C	0.0509 (13)	−0.0946 (15)	0.4387 (8)	0.066 (7)*	0.172 (3)
H11C	0.0340	−0.1778	0.4637	0.099*	0.172 (3)
H12C	0.0027	−0.0352	0.4166	0.099*	0.172 (3)
H13C	0.0907	−0.0349	0.4731	0.099*	0.172 (3)
C1B	−0.05882 (9)	0.53181 (16)	0.34180 (8)	0.0342 (3)	
H11B	−0.0275	0.5864	0.3137	0.051*	
H12B	−0.0330	0.5441	0.3932	0.051*	
H13B	−0.1153	0.5689	0.3310	0.051*	
N1B	−0.05977 (7)	0.37513 (12)	0.32293 (7)	0.0251 (3)	
H1B	−0.0076 (10)	0.3408 (17)	0.3347 (8)	0.030*	
H2B	−0.0796 (9)	0.3654 (17)	0.2750 (9)	0.030*	
C2B	−0.11157 (9)	0.28503 (17)	0.35852 (9)	0.0365 (4)	
H21B	−0.1073	0.1819	0.3460	0.055*	
H22B	−0.1690	0.3167	0.3423	0.055*	
H23B	−0.0926	0.2970	0.4107	0.055*	

### Atomic displacement parameters (Å^2^)

**Table:**

	*U*^11^	*U*^22^	*U*^33^	*U*^12^	*U*^13^	*U*^23^
O1A	0.0194 (4)	0.0320 (5)	0.0310 (5)	0.0004 (4)	0.0034 (4)	0.0035 (4)
O2A	0.0232 (5)	0.0452 (6)	0.0406 (6)	−0.0027 (4)	−0.0006 (4)	−0.0006 (5)
O3A	0.0355 (6)	0.0370 (6)	0.0474 (7)	0.0063 (5)	0.0006 (5)	0.0110 (5)
O4A	0.0376 (6)	0.0310 (6)	0.0437 (6)	−0.0147 (4)	0.0011 (5)	0.0020 (5)
O5A	0.0393 (6)	0.0334 (6)	0.0339 (6)	−0.0018 (4)	−0.0004 (5)	0.0087 (5)
N1A	0.0243 (6)	0.0345 (7)	0.0278 (6)	0.0046 (5)	0.0065 (5)	−0.0006 (5)
N2A	0.0242 (5)	0.0283 (6)	0.0296 (6)	−0.0040 (5)	0.0076 (5)	0.0019 (5)
C1A	0.0201 (6)	0.0256 (7)	0.0194 (8)	0.0007 (5)	0.0084 (5)	−0.0024 (5)
C2A	0.0217 (7)	0.0243 (7)	0.0247 (9)	−0.0007 (5)	0.0095 (6)	−0.0047 (6)
C3A	0.0262 (7)	0.0237 (7)	0.0264 (9)	0.0017 (5)	0.0102 (6)	−0.0006 (6)
C4A	0.0207 (6)	0.0303 (7)	0.0223 (7)	0.0034 (5)	0.0054 (5)	−0.0013 (5)
C5A	0.0189 (6)	0.0295 (7)	0.0229 (7)	−0.0033 (5)	0.0069 (5)	−0.0037 (5)
C6A	0.0240 (6)	0.0236 (6)	0.0242 (7)	−0.0009 (5)	0.0092 (5)	−0.0003 (5)
C7A	0.0232 (8)	0.0256 (9)	0.0324 (10)	−0.0048 (6)	0.0057 (7)	−0.0015 (7)
C8A	0.0333 (12)	0.0502 (13)	0.0409 (13)	−0.0134 (11)	0.0153 (10)	−0.0074 (12)
C9A	0.0350 (9)	0.0255 (9)	0.0377 (10)	−0.0074 (7)	0.0089 (8)	−0.0044 (7)
C10A	0.0408 (13)	0.0430 (13)	0.0348 (13)	−0.0144 (11)	0.0114 (11)	−0.0094 (10)
O1C	0.0194 (4)	0.0320 (5)	0.0310 (5)	0.0004 (4)	0.0034 (4)	0.0035 (4)
O2C	0.0232 (5)	0.0452 (6)	0.0406 (6)	−0.0027 (4)	−0.0006 (4)	−0.0006 (5)
O3C	0.0355 (6)	0.0370 (6)	0.0474 (7)	0.0063 (5)	0.0006 (5)	0.0110 (5)
O4C	0.0376 (6)	0.0310 (6)	0.0437 (6)	−0.0147 (4)	0.0011 (5)	0.0020 (5)
O5C	0.0393 (6)	0.0334 (6)	0.0339 (6)	−0.0018 (4)	−0.0004 (5)	0.0087 (5)
N1C	0.0243 (6)	0.0345 (7)	0.0278 (6)	0.0046 (5)	0.0065 (5)	−0.0006 (5)
N2C	0.0242 (5)	0.0283 (6)	0.0296 (6)	−0.0040 (5)	0.0076 (5)	0.0019 (5)
C1C	0.0201 (6)	0.0256 (7)	0.0194 (8)	0.0007 (5)	0.0084 (5)	−0.0024 (5)
C2C	0.0217 (7)	0.0243 (7)	0.0247 (9)	−0.0007 (5)	0.0095 (6)	−0.0047 (6)
C3C	0.0262 (7)	0.0237 (7)	0.0264 (9)	0.0017 (5)	0.0102 (6)	−0.0006 (6)
C4C	0.0207 (6)	0.0303 (7)	0.0223 (7)	0.0034 (5)	0.0054 (5)	−0.0013 (5)
C5C	0.0189 (6)	0.0295 (7)	0.0229 (7)	−0.0033 (5)	0.0069 (5)	−0.0037 (5)
C6C	0.0240 (6)	0.0236 (6)	0.0242 (7)	−0.0009 (5)	0.0092 (5)	−0.0003 (5)
C1B	0.0378 (8)	0.0301 (7)	0.0324 (8)	0.0000 (6)	0.0046 (6)	−0.0019 (6)
N1B	0.0198 (5)	0.0283 (6)	0.0271 (6)	0.0006 (4)	0.0057 (5)	0.0006 (5)
C2B	0.0300 (7)	0.0399 (8)	0.0411 (9)	−0.0055 (6)	0.0117 (6)	0.0058 (7)

### Geometric parameters (Å, °)

**Table:**

O1A—C1A	1.2770 (16)	C10A—H13A	0.9800
O2A—N1A	1.2375 (15)	C1C—C2C	1.455 (2)
O3A—N1A	1.2339 (15)	C2C—C3C	1.369 (2)
O4A—N2A	1.2326 (15)	C2C—C7C	1.524 (3)
O5A—N2A	1.2318 (15)	C3C—H31C	0.9500
N1A—C4A	1.4440 (17)	C7C—C9C	1.514 (3)
N2A—C6A	1.4546 (17)	C7C—C8C	1.547 (4)
C1A—C6A	1.4412 (18)	C7C—H7C	1.0000
C1A—C2A	1.4493 (19)	C8C—H81C	0.9800
C2A—C3A	1.3700 (19)	C8C—H82C	0.9800
C2A—C7A	1.5269 (19)	C8C—H83C	0.9800
C3A—C4A	1.4073 (19)	C9C—C10C	1.520 (4)
C3A—H31A	0.9500	C9C—H91C	0.9900
C4A—C5A	1.3758 (19)	C9C—H92C	0.9900
C5A—C6A	1.3830 (18)	C10C—H11C	0.9800
C5A—H51A	0.9500	C10C—H12C	0.9800
C7A—C9A	1.525 (2)	C10C—H13C	0.9800
C7A—C8A	1.549 (4)	C1B—N1B	1.4771 (18)
C7A—H71A	1.0000	C1B—H11B	0.9800
C8A—H81A	0.9800	C1B—H12B	0.9800
C8A—H82A	0.9800	C1B—H13B	0.9800
C8A—H83A	0.9800	N1B—C2B	1.4845 (18)
C9A—C10A	1.521 (3)	N1B—H1B	0.904 (16)
C9A—H91A	0.9900	N1B—H2B	0.902 (17)
C9A—H92A	0.9900	C2B—H21B	0.9800
C10A—H11A	0.9800	C2B—H22B	0.9800
C10A—H12A	0.9800	C2B—H23B	0.9800
			
O3A—N1A—O2A	122.63 (12)	C9C—C7C—C8C	112.1 (3)
O3A—N1A—C4A	118.58 (11)	C2C—C7C—C8C	111.4 (3)
O2A—N1A—C4A	118.79 (12)	C9C—C7C—H7C	106.5
O5A—N2A—O4A	122.53 (12)	C2C—C7C—H7C	106.5
O5A—N2A—C6A	119.53 (11)	C8C—C7C—H7C	106.5
O4A—N2A—C6A	117.93 (11)	C7C—C8C—H81C	109.5
O1A—C1A—C6A	123.52 (12)	C7C—C8C—H82C	109.5
O1A—C1A—C2A	121.27 (11)	H81C—C8C—H82C	109.5
C6A—C1A—C2A	115.20 (11)	C7C—C8C—H83C	109.5
C3A—C2A—C1A	120.79 (12)	H81C—C8C—H83C	109.5
C3A—C2A—C7A	120.62 (13)	H82C—C8C—H83C	109.5
C1A—C2A—C7A	118.59 (12)	C7C—C9C—C10C	113.5 (3)
C2A—C3A—C4A	120.73 (13)	C7C—C9C—H91C	108.9
C2A—C3A—H31A	119.6	C10C—C9C—H91C	108.9
C4A—C3A—H31A	119.6	C7C—C9C—H92C	108.9
C5A—C4A—C3A	121.43 (12)	C10C—C9C—H92C	108.9
C5A—C4A—N1A	118.98 (12)	H91C—C9C—H92C	107.7
C3A—C4A—N1A	119.49 (12)	C9C—C10C—H11C	109.5
C4A—C5A—C6A	118.36 (12)	C9C—C10C—H12C	109.5
C4A—C5A—H51A	120.8	H11C—C10C—H12C	109.5
C6A—C5A—H51A	120.8	C9C—C10C—H13C	109.5
C5A—C6A—C1A	123.42 (12)	H11C—C10C—H13C	109.5
C5A—C6A—N2A	116.20 (11)	H12C—C10C—H13C	109.5
C1A—C6A—N2A	120.23 (11)	N1B—C1B—H11B	109.5
C9A—C7A—C2A	111.19 (13)	N1B—C1B—H12B	109.5
C9A—C7A—C8A	111.13 (16)	H11B—C1B—H12B	109.5
C2A—C7A—C8A	111.02 (16)	N1B—C1B—H13B	109.5
C9A—C7A—H71A	107.8	H11B—C1B—H13B	109.5
C2A—C7A—H71A	107.8	H12B—C1B—H13B	109.5
C8A—C7A—H71A	107.8	C1B—N1B—C2B	113.36 (12)
C10A—C9A—C7A	112.75 (19)	C1B—N1B—H1B	109.0 (10)
C10A—C9A—H91A	109.0	C2B—N1B—H1B	109.6 (10)
C7A—C9A—H91A	109.0	C1B—N1B—H2B	109.0 (10)
C10A—C9A—H92A	109.0	C2B—N1B—H2B	107.9 (10)
C7A—C9A—H92A	109.0	H1B—N1B—H2B	107.9 (14)
H91A—C9A—H92A	107.8	N1B—C2B—H21B	109.5
C3C—C2C—C1C	120.69 (19)	N1B—C2B—H22B	109.5
C3C—C2C—C7C	122.7 (2)	H21B—C2B—H22B	109.5
C1C—C2C—C7C	116.4 (2)	N1B—C2B—H23B	109.5
C2C—C3C—H31C	119.2	H21B—C2B—H23B	109.5
C9C—C7C—C2C	113.3 (3)	H22B—C2B—H23B	109.5
			
O1A—C1A—C2A—C3A	−179.26 (17)	O1A—C1A—C6A—N2A	2.4 (3)
C6A—C1A—C2A—C3A	1.1 (2)	C2A—C1A—C6A—N2A	−177.99 (13)
O1A—C1A—C2A—C7A	1.9 (2)	O5A—N2A—C6A—C5A	154.19 (12)
C6A—C1A—C2A—C7A	−177.74 (16)	O4A—N2A—C6A—C5A	−24.80 (17)
C1A—C2A—C3A—C4A	−0.7 (2)	O5A—N2A—C6A—C1A	−30.0 (2)
C7A—C2A—C3A—C4A	178.17 (17)	O4A—N2A—C6A—C1A	151.04 (15)
C2A—C3A—C4A—C5A	1.4 (2)	C3A—C2A—C7A—C9A	−60.4 (2)
C2A—C3A—C4A—N1A	177.89 (13)	C1A—C2A—C7A—C9A	118.50 (17)
O3A—N1A—C4A—C5A	177.35 (12)	C3A—C2A—C7A—C8A	63.9 (2)
O2A—N1A—C4A—C5A	−2.18 (18)	C1A—C2A—C7A—C8A	−117.23 (18)
O3A—N1A—C4A—C3A	0.8 (2)	C2A—C7A—C9A—C10A	−61.0 (2)
O2A—N1A—C4A—C3A	−178.72 (14)	C8A—C7A—C9A—C10A	174.80 (18)
C3A—C4A—C5A—C6A	−2.7 (2)	C3C—C2C—C7C—C9C	40.3 (6)
N1A—C4A—C5A—C6A	−179.14 (11)	C1C—C2C—C7C—C9C	−144.7 (5)
C4A—C5A—C6A—C1A	3.3 (2)	C3C—C2C—C7C—C8C	−87.2 (7)
C4A—C5A—C6A—N2A	178.96 (11)	C1C—C2C—C7C—C8C	87.8 (7)
O1A—C1A—C6A—C5A	177.91 (15)	C2C—C7C—C9C—C10C	66.4 (11)
C2A—C1A—C6A—C5A	−2.5 (2)	C8C—C7C—C9C—C10C	−166.5 (11)

### Hydrogen-bond geometry (Å, °)

**Table:**

*D*—H···*A*	*D*—H	H···*A*	*D*···*A*	*D*—H···*A*
N1B—H1B···O1A	0.904 (16)	1.914 (16)	2.7585 (15)	154.7 (14)
N1B—H2B···O1A^i^	0.902 (17)	1.868 (17)	2.7173 (16)	156.2 (15)

Symmetry codes:  (i) −*x*, *y*, −*z*+1/2.
